# Association Between Serum Potassium Levels and Cardiac Arrest Risk in Emergency Resuscitation Room Patients: A Retrospective Cohort Study

**DOI:** 10.3390/jcm15145733

**Published:** 2026-07-22

**Authors:** Yongkai Li, Xialaibaitigu Saimaiti, Yingping Tian, Hengbo Gao, Jianzhong Yang

**Affiliations:** 1 Emergency Department, The Second Hospital of Hebei Medical University, Shijiazhuang 050000, China; 29405814@hebmu.edu.cn; 2 Emergency Trauma Center, The Fifth Affiliated Hospital of Xinjiang Medical University, Urumqi 830000, China; x13079955250@163.com; 3 Emergency Trauma Center, The First Affiliated Hospital of Xinjiang Medical University, Urumqi 830000, China

**Keywords:** cardiac arrest, potassium, hypokalemia, hyperkalemia, risk prediction, emergency medicine, resuscitation

## Abstract

**Background:** This study aimed to investigate the association between serum potassium levels and the risk of cardiac arrest in patients admitted to the emergency resuscitation room and to identify the optimal potassium range associated with the lowest risk. **Methods:** We conducted a retrospective cohort study of 784 adults (384 with cardiac arrest and 400 controls) admitted to the emergency resuscitation room of a tertiary academic medical center between January 2020 and July 2021. Using multivariable logistic regression and restricted cubic spline analysis with four knots, we examined the nonlinear relationship between serum potassium levels and cardiac arrest risk after adjusting for critical confounders including lactate levels and MEWS scores. **Results:** A robust J-shaped association was observed between serum potassium levels and cardiac arrest risk. The lowest risk occurred within the range of 3.1–4.0 mmol/L, with significantly increased risk both below and above this range. The association remained significant after adjustment for lactate alone (*p*-nonlinear < 0.001) and after simultaneous adjustment for the MEWS score and lactate (*p*-nonlinear < 0.05). Serum potassium demonstrated fair predictive accuracy for cardiac arrest with an AUC of 0.709 (95% CI: 0.672–0.747). At the optimal cut-off value of 4.45 mmol/L, specificity reached 87.5% and positive predictive value was 80.4%. **Conclusions:** This study reveals a consistent J-shaped distribution between serum potassium levels and cardiac arrest risk in emergency resuscitation room patients, with the safest range identified at 3.1–4.0 mmol/L. These findings suggest that maintaining potassium levels in the lower physiological range may be beneficial for preventing cardiac arrest in critically ill emergency patients. Serum potassium assessment provides a valuable tool for risk stratification in resuscitation settings.

## 1. Introduction

Cardiac arrest (CA) is characterized by the abrupt cessation of cardiac activity, resulting in unresponsiveness and absence of normal breathing or discernible circulation [1]. Out-of-hospital cardiac arrest (OHCA) and in-hospital cardiac arrest (IHCA) represent critical threats to global human health and survival. Together, they constitute a major public health challenge worldwide [2]. The etiologies of cardiac arrest are complex and multifactorial, among which electrolyte disturbances—particularly potassium (K^+^) dysregulation—represent a significant and modifiable risk factor. Potassium is the most abundant cation in the human body and is critical for normal cellular function. The normal reference range for serum potassium concentration in clinical laboratories is 3.5–5.5 mmol/L. Hyperkalemia is defined as a serum potassium level exceeding 5.5 mmol/L, whereas hypokalemia is defined as a level below 3.5 mmol/L [3]. The gradient of potassium ions across cell membranes is essential for normal neuromuscular and cardiac physiological functions. Potassium plays a key role in maintaining cellular metabolism, regulating osmotic pressure and acid-base balance, preserving neuromuscular excitability, and supporting myocardial automaticity, conductivity, and excitability [4]. Alterations in potassium homeostasis can lead to dysfunction in the neuromuscular and gastrointestinal systems, as well as impair cardiac performance [5]. Alterations in potassium homeostasis have been implicated in the pathophysiology of myocardial infarction, heart failure, and renal failure. Current evidence predominantly highlights hyperkalemia and hypokalemia as established risk factors for cardiac arrest. Nevertheless, the precise serum potassium thresholds associated with a graded increase in risk—as well as the exact concentration ranges that predict arrest—have not been fully elucidated. According to the ACC/AHA guidelines, serum potassium level above 4.0 mEq/L is recommended for AMI patients; in some circumstances, a target of 4.5–5.0 mEq/L may be considered. This recommendation stems from the observation that levels below 3.5 mEq/L are linked to a heightened risk of ventricular dysrhythmias [6,7,8]. However, these recommendations are primarily derived from cardiovascular populations, particularly those with AMI, and their applicability to heterogeneous, critically ill patients in the emergency resuscitation room remains uncertain. There is currently a lack of high-quality evidence defining the optimal serum potassium range associated with cardiac arrest risk in this broad and high-risk population. Therefore, this study seeks to characterize the relationship between serum potassium levels and the occurrence of cardiac arrest in the emergency resuscitation room and to assess its utility as a predictive biomarker for imminent cardiac arrest, with the ultimate objective of informing evidence-based strategies for potassium management in critically ill patients.

## 2. Information and Methods

### 2.1. Study Subjects

Inclusion criteria: (1) age ≥18 years; (2) meeting the diagnostic criteria for cardiac arrest in the guidelines for cardiopulmonary resuscitation [1]. Exclusion criteria: (1) cardiac arrest occurring before admission and outside the hospital; (2) with malignant tumors or other diseases in the terminal stage; (3) with life-threatening trauma; 4) missing essential clinical data.

### 2.2. Ethical Approval

The Ethics Committee of the First Affiliated Hospital of Xinjiang Medical University (K202301-27) approved this study. All procedures were performed in accordance with the Declaration of Helsinki [9] and applicable institutional guidelines. Informed consent was not required, as the study involved no prospective interventions and relied solely on retrospective data.

### 2.3. Data Collection

Data collection was performed via the hospital’s outpatient medical records system. The following baseline variables were obtained upon emergency room admission: patients’ age and sex; ethnicity (Han/Uyghur/Kazakh/other); the MEWS, stratified as <5 or ≥5; and vital sign measurements, including body temperature, heart rate, SBP, MAP, and respiratory rate, intubation status, consciousness level, symptoms, medical history, and various lab results (blood routine, biochemical parameters, blood gas analysis, coagulation indices, inflammatory factors, cardiac markers).

### 2.4. Statistical Methods

Statistical analysis and data visualization were conducted using R language (v.4.2.0), GraphPad Prism (v.9.0), and MedCalc (v.20.0) software. Continuous variables with non-normal distributions were presented as median and interquartile range and compared using the Mann–Whitney U test. Categorical variables were denoted as frequencies and percentages, with chi-square test or Fisher’s exact test applied for between-group comparisons. To mitigate multicollinearity among predictor variables, least absolute shrinkage and selection operator (LASSO) regression with 10-fold cross-validation was employed for variable selection. Significant variables identified from the LASSO regression, along with clinically relevant factors, were subsequently included in a multivariable logistic regression model (using stepwise selection with a significance level of *p* < 0.05 for entry) to identify independent factors associated with cardiac arrest. Restricted cubic spline (RCS) analysis with 4 knots was then applied based on the logistic regression model to visualize the potential nonlinear relationship between serum potassium (K^+^) levels and the log-odds of cardiac arrest. A two-sided *p*-value < 0.05 was considered statistically significant.

## 3. Results

### 3.1. Study Population

**Figure 1:**
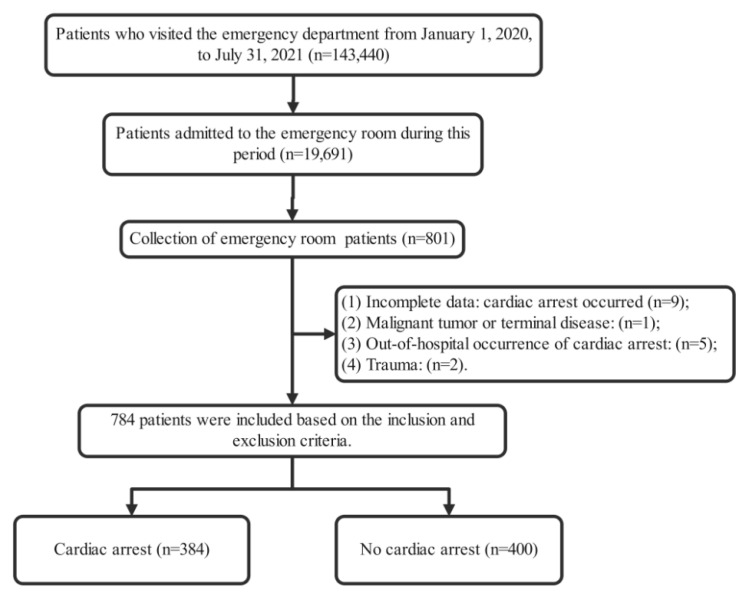
Flow chart for developing and validating predictive models.

The flow diagram is illustrated in Figure 1.

### 3.2. General Information

We screened all emergency department visits between 1 January 2020 and 31 July 2021, identifying 143,440 patients during this period. Of these, 19,691 required admission to the resuscitation room. Cardiac arrest occurred in 401 of these patients (2.04%), and after rigorous application of our eligibility criteria, 384 arrest cases met the requirements for study inclusion. In addition, 400 patients who did not experience cardiac arrest were randomly selected from the emergency resuscitation room. Ultimately, 784 emergency patients were enrolled, including 384 with cardiac arrest and 400 without cardiac arrest. The patients with cardiac arrest had a median age of 62 years (49.00 to 76.00); 264 (68.8%) were male and 120 (31.2%) were female. The patients without cardiac arrest had a median age of 63 years (50.00 to 77.00); 232 (58.0%) were male and 168 (42.0%) were female (Supplementary Table S1).

### 3.3. Variable Selection via LASSO and Multivariable Logistic Regression

**Figure 2:**
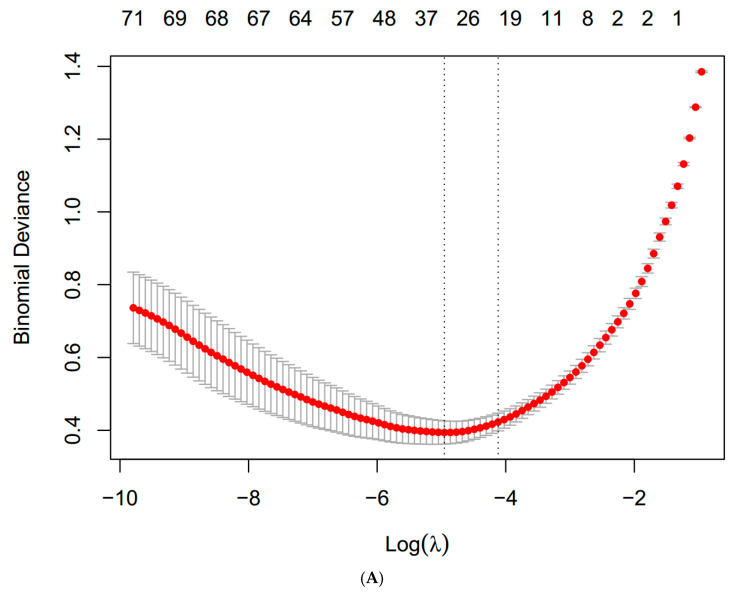

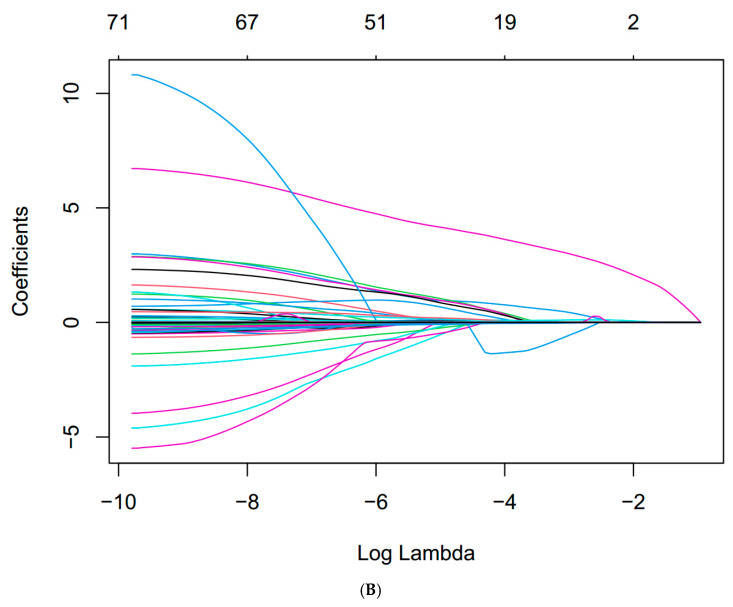
Using the LASSO regression model for variable selection. (**A**) shows the tuning-path for the optimal penalty parameter λ in the LASSO model. Ten-fold cross-validation was applied, with the left dashed line indicating λmin (the value yielding the minimum cross-validated error) and the right dashed line denoting λmin plus 1 standard error, at which point the most parsimonious set of predictors was retained. (**B**) depicts the coefficient trajectories of all 73 candidate variables across the entire range of λ.

**Figure 3:**
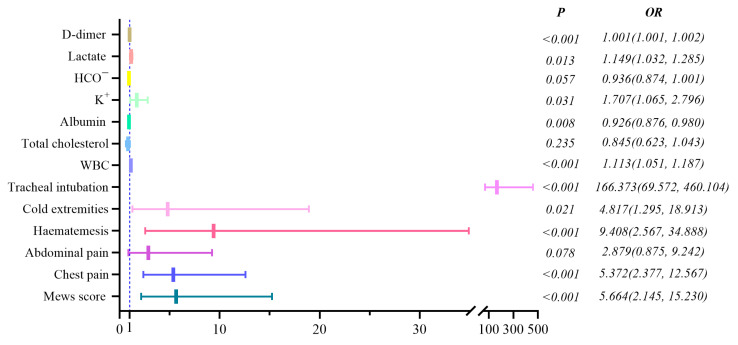
Predictors of cardiac arrest in the emergency room (multivariate analysis).

Variable selection was accomplished using LASSO regression with 10-fold cross-validation, where the penalty parameter λ was optimized at the minimum binomial deviance (Figure 2A). As λ increased, the regression coefficients of the initial 73 candidate variables were penalized toward zero; several were eliminated entirely (Figure 2B), effectively preventing overfitting and retaining only the most informative features. These retained variables were subsequently tested in a multivariable logistic model (*p* < 0.05), ultimately yielding 13 independent risk factors: chest pain, MEWS score, abdominal pain, Tracheal intubation, hematemesis, cold extremities, WBC, albumin, potassium, HCO^−^, lactate, and D-dimer (Table 1 and Figure 3).

**Figure 4:**
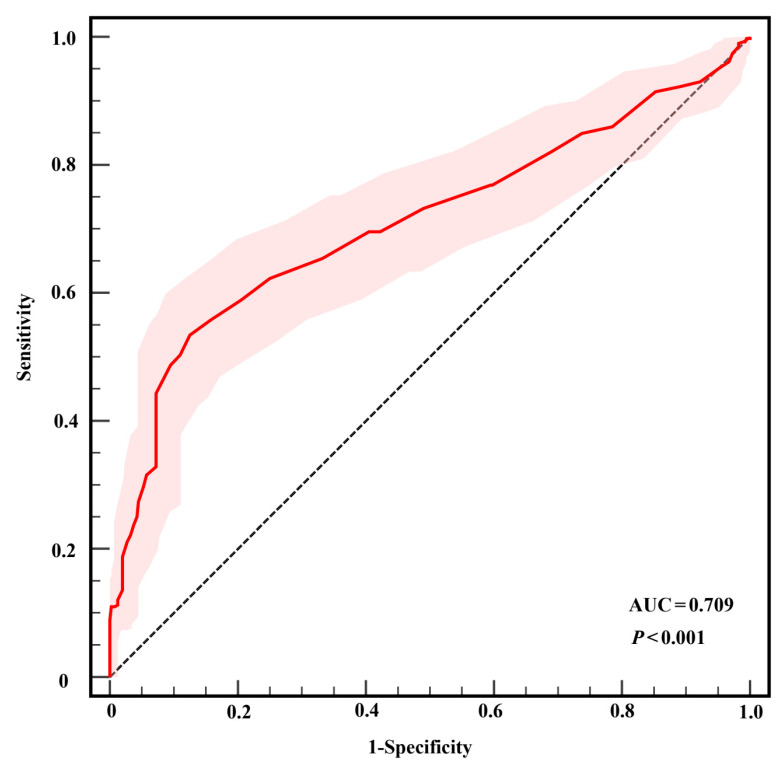
ROC curve of serum potassium level for predicting cardiac arrest.

Serum potassium showed fair predictive performance for cardiac arrest, with an AUC of 0.709 (95% CI 0.672–0.747). At the optimal cut-off of 4.45 mmol/L, the test yielded a sensitivity of 0.534 (95% CI 0.484–0.584) and a specificity of 0.875 (95% CI 0.843–0.907). The corresponding PPV and NPV were 0.804 (95% CI 0.755–0.853) and 0.662 (95% CI 0.621–0.702), respectively (Table 2, Figure 4).

**Figure 5:**
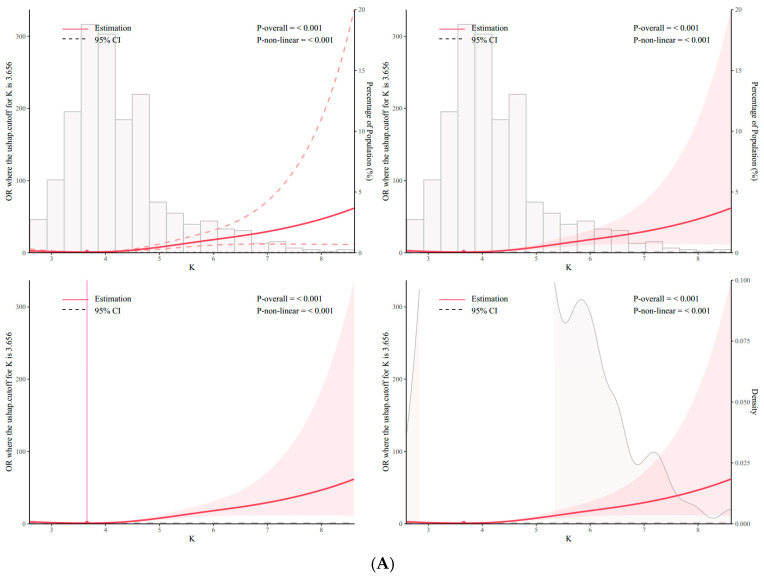

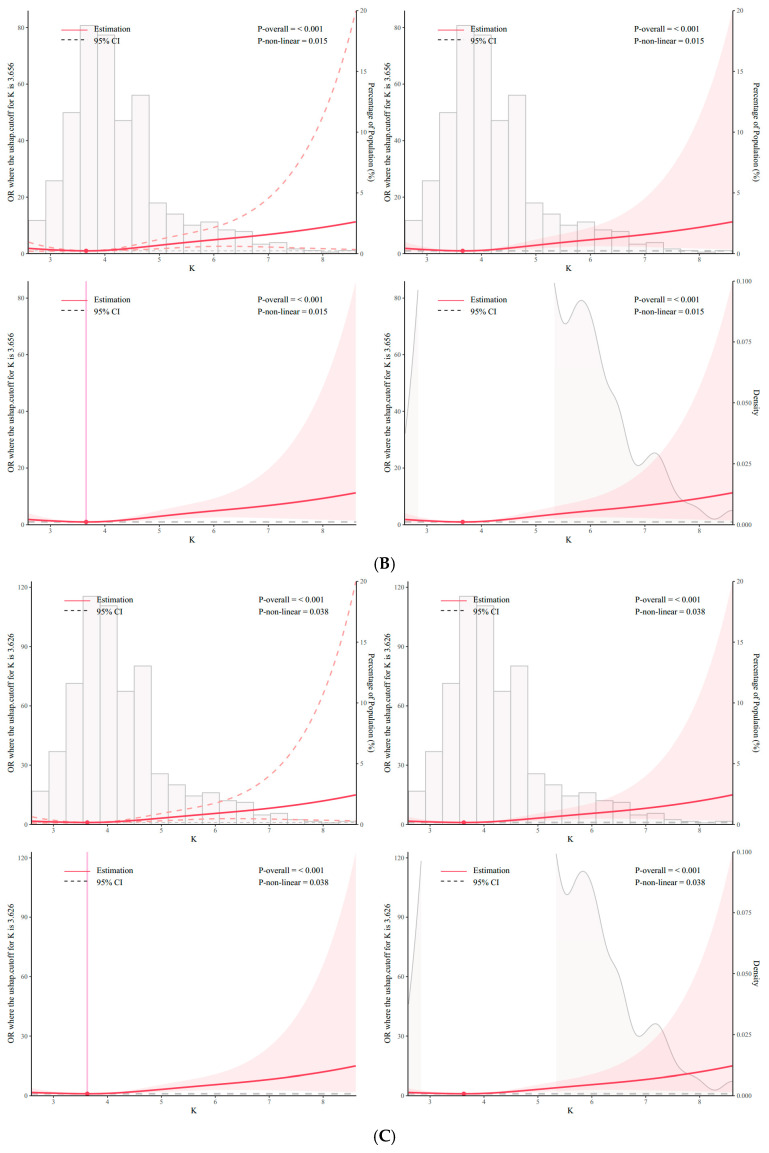
(**A**) J-shaped association between serum potassium and cardiac arrest risk. (**B**) J-shaped association between serum potassium and cardiac arrest risk after adjustment for lactate. (**C**) J-shaped association between serum potassium and cardiac arrest risk after simultaneous adjustment for MEWS score and lactate.

In analyzing the association between serum potassium levels and the risk of cardiac arrest, we observed a robust nonlinear J-shaped distribution. In the unadjusted analysis, serum potassium demonstrated a significant J-shaped association with cardiac arrest risk (*p*-overall < 0.001; *p*-non-linear < 0.001), with the lowest risk observed within the range of 3.2 to 4.0 mmol/L. This J-shaped association remained significant after adjustment for lactate levels alone, as well as after simultaneous adjustment for MEWS score and lactate levels (all *p*-overall < 0.001; *p*-non-linear < 0.05), with the nadir of risk consistently lying within the range of 3.1 to 4.0 mmol/L. Cardiac arrest risk increased progressively both below this range and above 4.0 mmol/L (Figure 5A–C).

## 4. Discussion

Maintenance of potassium homeostasis is critical for normal myocardial electrophysiology. Hypokalemia promotes malignant arrhythmias (such as ventricular tachycardia and fibrillation) by inducing hyperpolarization and enhanced automaticity of cardiac myocytes. Conversely, hyperkalemia leads to depolarization, impaired electrical conduction, conduction block, and myocardial suppression, potentially progressing to asystole or pulseless electrical activity (PEA), resulting in extensive cardiomyocyte injury or electrical dysfunction [10]. Given that potassium dysregulation is a well-established reversible cause of cardiac arrest, clarifying its quantitative distribution with CA risk and defining optimal management targets are of major importance for precise risk mitigation in the emergency resuscitation setting. However, a clear consensus regarding the optimal potassium range for this specific high-risk population remains lacking.

In this retrospective analysis of 784 patients in the emergency resuscitation room, we identified for the first time a J-shaped nonlinear association between serum potassium levels and in-hospital cardiac arrest. In the unadjusted model, the lowest risk of cardiac arrest was observed at potassium levels between 3.2 and 4.0 mmol/L. After adjustment for arterial lactate levels measured by blood gas analysis, the nadir of risk remained within the range of 3.2 to 4.0 mmol/L. Further adjustment for both lactate and the MEWS score at the emergency resuscitation room showed the lowest risk occurring within a range of 3.1 to 4.0 mmol/L. Across multiple models, the optimal risk window remained consistently stable between 3.1 and 4.0 mmol/L. These findings provide important preliminary evidence to guide potassium management strategies in the emergency department.

Our findings are partially consistent with those reported by Goyal et al. [11] in a large-scale JAMA study of AMI patients. That study also described a U-shaped distribution between serum potassium and in-hospital mortality; however, the optimal range associated with the lowest mortality (3.5–4.5 mEq/L) was somewhat higher than that identified in our analysis. In their cohort of 38,689 patients with AMI, both admission and in-hospital mean potassium levels exhibited a U-shaped association with in-hospital mortality. After multivariable adjustment, the lowest mortality risk (4.8%) was observed at potassium levels of 3.5–4.0 mmol/L (reference group). Compared with the reference, mortality risk was similar at 4.0–<4.5 mmol/L (OR = 1.19), doubled at 4.5–<5.0 mmol/L (OR = 1.99), and increased further at higher levels. Hypokalemia (<3.5 mmol/L) was also associated with elevated mortality. The incidence of ventricular arrhythmias or cardiac arrest remained relatively stable within the potassium range of 3.0–<5.0 mmol/L, rising significantly only below 3.0 mmol/L or at levels ≥5.0 mmol/L. Furthermore, serum potassium levels demonstrated strong predictive value for neurological outcomes following cardiac arrest. A prospective, multicenter study [12] found that among patients with cardiac-derived OHCA who achieved return of spontaneous circulation (ROSC), serum potassium levels were significantly associated with favorable neurological outcomes, with the proportion of favorable outcomes decreasing as potassium levels increased. Patients were stratified into four quartiles based on serum potassium levels: Q1 (K^+^ ≤ 3.8 mmol/L), Q2 (3.8 < K^+^ ≤ 4.5 mmol/L), Q3 (4.5 < K^+^ ≤ 5.6 mmol/L), and Q4 (K^+^ > 5.6 mmol/L). The highest rate of favorable neurological outcomes was observed in Q1 (44.8%), followed by Q2 (30.3%), Q3 (11.7%), and Q4 (4.5%) (*p* < 0.001). In multivariable analysis, higher serum potassium levels remained independently associated with a decreased likelihood of favorable neurological outcome (*p* < 0.001). In a retrospective observational study examining the association between serum potassium levels at intensive care unit (ICU) admission and functional outcomes in comatose survivors of cardiac arrest, the reference range for normokalemia was defined as 3.0–4.9 mmol/L. One in five patients exhibited dyskalemia at ICU presentation. Hyperkalemia (≥5.0 mmol/L) was independently associated with unfavorable functional status at 180 days, whereas hypokalemia (<3.0 mmol/L) showed no significant association. These findings suggest that prompt identification and correction of hyperkalemia may represent a modifiable determinant of long-term neurologic recovery [13]. In OHCA patients, serum potassium levels at admission were strongly associated with survival outcomes. Although Choi et al. [14] found that hypokalemia was inversely associated with good functional outcome in contrast to normokalemia group (K ^+^ = 3.5–5.4 meq/L), this association may be influenced by confounding factors; Patients in the hypokalemic group had more favorable resuscitation conditions (such as a higher rate of shockable rhythm, more bystander CPR, and public arrest), which may be responsible for the real improvement in functional outcomes. In contrast, hyperkalemia was independently associated with reduced survival to hospital discharge but did not significantly affect functional outcomes.

Current clinical practice guidelines recommend maintaining serum potassium levels between 4.0 and 5.0 mmol/L in patients with acute myocardial infarction (AMI), a target largely derived from small-scale studies [6,8]. However, recent evidence suggests a U-shaped association between in-hospital mean potassium levels and mortality among AMI inpatients, with the lowest mortality observed in those with potassium levels between 3.5 and <4.5 mmol/L [11]. A meta-analysis examining both short- and long-term mortality [15] demonstrated significantly increased risks among AMI patients with serum potassium concentrations <3.5 mEq/L, 4.5–<5.0 mEq/L, and ≥5.0 mEq/L. Furthermore, potassium levels <3.5 mEq/L were significantly associated with ventricular arrhythmias. The analysis suggested an inverse relationship between hypokalemic potassium concentrations and both short- and long-term mortality, as well as the incidence of ventricular arrhythmias, following AMI. There is also evidence indicating adverse outcomes associated with serum potassium levels ≥4.5 mEq/L. Due to heterogeneity among existing studies, further research is needed to determine whether current guideline recommendations should be revised. Nonetheless, the present study, along with findings from Goyal [11] and the recent meta-analysis [15], consistently indicates that serum potassium levels ≥4.5 mmol/L may be associated with increased risk of adverse outcomes. This accumulating evidence underscores the need to re-evaluate the optimal potassium target range in contemporary AMI management guidelines.

## 5. Limitations and Future Directions

This study has several limitations. Its single-center, retrospective observational design inherently limits causal inference. Although adjustments were made for arterial lactate and the MEWS score, residual confounding cannot be fully excluded. The lack of continuous potassium monitoring data and limited power in subgroup analyses further constrain the generalizability of our findings. Therefore, the results are not yet sufficient to directly inform clinical guideline revisions.

Future research should prioritize the following directions:

(1) Employ continuous or high-frequency potassium monitoring to characterize dynamic potassium trajectories and their predictive utility;
(2) Conduct well-designed, adequately powered randomized controlled trials to evaluate the effect of potassium-specific interventions on in-hospital cardiac arrest;
(3) Establish large prospective multicenter cohorts with stratification based on key comorbidities to define individualized optimal potassium target ranges.

## 6. Conclusions

In summary, this study demonstrates a J-shaped association between serum potassium levels and the risk of cardiac arrest in patients treated in the emergency resuscitation room. Although the optimal range requires further clarification, our data suggest that maintaining potassium levels near the lower end of the physiological range (3.5–4.0 mmol/L) may be relatively safe. Initial potassium levels ≤3.2 mmol/L or >4.0 mmol/L—particularly those exceeding 4.5 mmol/L—were significantly associated with an increased near-term risk of cardiac arrest. Serum potassium may serve as a simple and effective biomarker for risk stratification in the emergency resuscitation room. However, prospective studies are needed to validate its utility as an interventional target and to establish specific treatment thresholds.

## Figures and Tables

**Table 1:** Multivariable logistic regression analysis for cardiac arrest risk.

Characteristic	OR (95% CI)	*p* Value
Intercept	0.093 (0.003, 2.310)	0.155
Mews Score	5.664 (2.145, 15.230)	<0.001
Chest pain	5.372 (2.377, 12.567)	<0.001
Abdominal pain	2.879 (0.875, 9.242)	0.078
Haematemesis	9.408 (2.567, 34.888)	<0.001
Cold extremities	4.817 (1.295, 18.913)	0.021
Tracheal intubation	166.373 (69.572, 460.104)	<0.001
WBC	1.113 (1.051, 1.187)	<0.001
Total cholesterol	0.845 (0.623, 1.043)	0.235
Albumin	0.926 (0.876, 0.980)	0.008
K^+^	1.707 (1.065, 2.796)	0.031
HCO^−^	0.936 (0.874, 1.001)	0.057
Lactate	1.149 (1.032, 1.285)	0.013
D-dimer	1.001 (1.001, 1.002)	<0.001

**Table 2:** Diagnostic accuracy of serum potassium for cardiac arrest.

Characteristic	AUC (95%Cl)	Sensitivity (95%Cl)	Specificity (95%Cl)	Cut-Off Value	PPV	NPV
K^+^	0.709 (0.672–0.747)	0.534 (0.484–0.584)	0.875 (0.843–0.907)	4.45	0.804 (0.755–0.853)	0.662 (0.621–0.702)

CI: confidence interval; PPV: positive predictive value; NPV: negative predictive value; AUC: area under the curve.

## Data Availability

The original contributions presented in this study are included in the article/Supplementary Material. Further inquiries can be directed to the corresponding authors.

## Supplementary Materials

The following supporting information can be downloaded at https://www.mdpi.com/article/10.3390/jcm15145733/s1, Table S1: Baseline characteristics of patients with and without cardiac arrest in the emergency resuscitation room; File S1: STROBE Statement—checklist of items that should be included in reports of observational studies.

## References

[1] Carrick R.T., Park J.G., McGinnes H.L., Lundquist C., Brown K.D., Janes W.A., Wessler B.S., Kent D.M. (2020). Clinical Predictive Models of Sudden Cardiac Arrest: A Survey of the Current Science and Analysis of Model Performances. J. Am. Heart Assoc..

[2] Jerkeman M., Sultanian P., Lundgren P., Nielsen N., Helleryd E., Dworeck C., Omerovic E., Nordberg P., Rosengren A., Hollenberg J. (2022). Trends in survival after cardiac arrest: A Swedish nationwide study over 30 years. Eur. Heart J..

[3] Palmer B.F., Carrero J.J., Clegg D.J., Colbert G.B., Emmett M., Fishbane S., Hain D.J., Lerma E., Onuigbo M., Rastogi A. (2021). Clinical Management of Hyperkalemia. Mayo. Clin. Proc..

[4] Kettritz R., Loffing J. (2023). Potassium homeostasis—Physiology and pharmacology in a clinical context. Pharmacol. Ther..

[5] Ferreira J.P., Butler J., Rossignol P., Pitt B., Anker S.D., Kosiborod M., Lund L.H., Bakris G.L., Weir M.R., Zannad F. (2020). Abnormalities of Potassium in Heart Failure: JACC State-of-the-Art Review. J. Am. Coll. Cardiol..

[6] Cohn J.N., Kowey P.R., Whelton P.K., Prisant L.M. (2000). New guidelines for potassium replacement in clinical practice: A contemporary review by the National Council on Potassium in Clinical Practice. Arch. Intern Med..

[7] Macdonald J.E., Struthers A.D. (2004). What is the optimal serum potassium level in cardiovascular patients?. J. Am. Coll. Cardiol..

[8] Antman E.M., Anbe D.T., Armstrong P.W., Bates E.R., Green L.A., Hand M., Hochman J.S., Krumholz H.M., Kushner F.G., Lamas G.A. (2004). ACC/AHA guidelines for the management of patients with ST-elevation myocardial infarction; A report of the American College of Cardiology/American Heart Association Task Force on Practice Guidelines (Committee to Revise the 1999 Guidelines for the Management of patients with acute myocardial infarction). J. Am. Coll. Cardiol..

[9] Millum J., Wendler D., Emanuel E.J. (2013). The 50th anniversary of the Declaration of Helsinki: Progress but many remaining challenges. Jama.

[10] Lott C., Truhlář A., Alfonzo A., Barelli A., González-Salvado V., Hinkelbein J., Nolan J.P., Paal P., Perkins G.D., Thies K.-C. (2021). European Resuscitation Council Guidelines 2021: Cardiac arrest in special circumstances. Resuscitation.

[11] Goyal A., Spertus J.A., Gosch K., Venkitachalam L., Jones P.G., Van den Berghe G., Kosiborod M. (2012). Serum potassium levels and mortality in acute myocardial infarction. Jama.

[12] Shida H., Matsuyama T., Iwami T., Okabayashi S., Yamada T., Hayakawa K., Yoshiya K., Irisawa T., Noguchi K., Nishimura T. (2020). Serum potassium level on hospital arrival and survival after out-of-hospital cardiac arrest: The CRITICAL study in Osaka, Japan. Eur. Heart J. Acute Cardiovasc. Care..

[13] Holm A., Lascarrou J.B., Cariou A., Reinikainen M., Laitio T., Kirkegaard H., Søreide E., Taccone F.S., Lääperi M., Skrifvars M.B. (2024). Potassium disorders at intensive care unit admission and functional outcomes after cardiac arrest. Resuscitation.

[14] Choi D.S., Shin S.D., Ro Y.S., Lee K.W. (2020). Relationship between serum potassium level and survival outcome in out-of-hospital cardiac arrest using CAPTURES database of Korea: Does hypokalemia have good neurological outcomes in out-of-hospital cardiac arrest?. Adv. Clin. Exp. Med..

[15] Colombo M.G., Kirchberger I., Amann U., Dinser L., Meisinger C. (2018). Association of serum potassium concentration with mortality and ventricular arrhythmias in patients with acute myocardial infarction: A systematic review and meta-analysis. Eur. J. Prev. Cardiol..

