# Supplementary material for: Association Between Serum Potassium Levels and Cardiac Arrest Risk in Emergency Resuscitation Room Patients: A Retrospective Cohort Study
Source: J Clin Med. 2026 Jul 22;15(14):5733. doi: 10.3390/jcm15145733 (PMC13413044; doi:10.3390/jcm15145733)
Supplement: Supplementary file 1 [file jcm-15-05733-s001.zip › jcm-4408556-Supplementary Table S1.pdf]

Supplementary Table S1. Baseline characteristics of patients with and without cardiac arrest in the emergency resuscitation room.

Characteristic		No cardiac arrest(n=400)	Cardiac arrest(n=384)	Z/x^2	*P*
Gender（n（%））∮	Male#	232(46.8)	264(53.2)	9.742	0.002
	Female#	168(58.3)	120(41.7)		
Age（years）		63(50~77)	62(49~76)	-1.003	0.316
**Race（n（%））**					
Han#		302(50.8)	292(49.2)	0.041	0.998
Uyghur#		54(51.4)	51(48.6)		
Kazakh#		10(52.6)	9(47.4)		
Other#		34(51.5)	32(48.5)		
**Mews Score（n（%））∮**					
＜5#		378(63.2)	220(36.8)	1419.890	＜0.001
≥5#		22(11.8)	164(88.2)		
**Vital signs**					
Temperature(℃)*∮		36.5(36.30~36.88)	36.7(36.30~36.90)	-3.471	0.001
Heart rate((beats/min)*∮		88(75~105)	102(88~117)	-6.719	＜0.001
SBP(mmHg)*∮		137.5(117~159)	121(100.00 ~132.75)	-8.901	＜0.001
MAP (mmHg)*∮		97(86~112)	88(74~96)	-8.118	＜0.001
Respiratory rate*∮		20(16~20)	20(18~21)	-3.776	＜0.001
Tracheal intubation#∮	No	392(81.7)	88(18.3)	465.243	＜0.001
	Yes	8(2.6)	296(97.4)		

**State of consciousness（n（%））∮**					
Alert#		380(62.8)	225(37.2)	149.871	＜0.001
Reacts to voice#		5(20.8)	19(79.2)		
Reacts to pain#		6(19.4)	25(80.6)		
Unresponsive#		9(7.3)	115(92.7)		
**Symptoms（n（%））**					
Hemoptysis#∮	No	386(50.5)	379(49.5)	4.002	0.045
	Yes	14(73.7)	5(26.3)		
Chest distress#∮	No	265(48.0)	287(52.0)	6.777	0.009
	Yes	135(58.2)	97(41.8)		
Dyspnea#∮	No	349(53.2)	307(46.8)	7.647	0.006
	Yes	51(39.8)	77(60.2)		
Palpitation#	No	369(51.9)	342(48.1)	2.357	0.125
	Yes	31(42.5)	42(57.5)		
Chest pain#	No	326 (51.4)	308(48.6)	0.211	0.646
	Yes	74(49.3)	76(50.7)		
Abdominal Pain#∮	No	385(53.5)	335(46.5)	21.217	＜0.001
	Yes	15(23.4)	49(76.6)		
Diarrhea#	No	391(51.0)	376(49.0)	0.026	0.873
	Yes	9(52.9)	8(47.1)		
Haematemesis#∮	No	392(52.2)	359(47.8)	9.885	0.002
	Yes	8(24.2)	25(75.8)		
Hematochezia#	No	381(51.5)	359(48.5)	1.146	0.284
	Yes	19(43.2)	25(56.8)		

Vomiting#∮	No	346(53.0)	307(47.0)	6.043	0.014
	Yes	54(41.2)	77(58.8)		
Oliguria#	No	382(51.2)	364(48.8)	0.213	0.644
	Yes	18(47.4)	20(52.6)		
Polyuria#	No	399(51.0)	384(49.0)	-	1.000
	Yes	1(100)	0(0)		
Hematuria#	No	399(51.2)	380(48.8)	0.890	0.346
	Yes	1(20)	4(80)		
Seizure#	No	394(51.0)	378(49.0)	0.005	0.943
	Yes	6(50)	6(50)		
Cold extremities#∮	No	392(56.2)	306(43.8)	67.274	＜0.001
	Yes	8(9.3)	78(90.7)		
**Medical history〔n（%）〕**					
Hypertension#	No	202(48.7)	213(51.3)	1.941	0.164
	Yes	198(53.7)	171(46.3)		
Diabetes#	No	296(49.2)	306(50.8)	3.555	0.059
	Yes	104(57.1)	78(42.9)		
Cardiac disease#	No	302(50.7)	294(49.3)	0.121	0.778
	Yes	98(52.1)	90(47.9)		
Renal disease#	No	384(50.7)	374(49.3)	1.191	0.275
	Yes	16(61.5)	10(38.5)		
**Blood routine examinations**					
WBC (10^9/L) *∮		7.60(5.65~9.98)	13.08(9.05~18.25)	-12.901	＜0.001
NEU (10^9/L) *∮		5.31(3.71~7.51)	9.86(5.77~15.27)	-10.734	＜0.001

LYM (10^9/L) *∮	1.22(0.78~1.72)	1.51(0.84~3.39)	-5.047	＜0.001
RBC (10^{12}/L) *∮	4.30(3.57~4.81)	3.94(2.82~4.77)	-3.860	＜0.001
HCT*∮	38.4(32.00~42.80)	36.8(26.20~43.40)	-2.512	0.012
HGB (g/L) *∮	129(106.25~145.00)	118(88~144)	-3.948	＜0.001
PLT (10^9/L）*∮	192.5(138.00~247.75)	162.5(98.50~243.00)	-3.781	＜0.001
**Biochemical parameters**				
BUN (mmol/L) *∮	6.25(4.52~8.91)	8.57(5.95~15.09)	-7.535	＜0.001
Cre (umol/L) *∮	68.34(52.22~95.58)	105.57(70.89~196.15)	-9.091	＜0.001
GLU (mmol/L) *∮	7.48(6.15~9.81)	9.66(6.59~14.74)	-5.699	＜0.001
Lactate (mmol/L) *∮	1.81(1.33~2.57)	6.63(3.22~11.38)	-17.599	＜0.001
CHO (mmol/L) *∮	3.8(3.00~4.63)	3.16(2.16~4.04)	-7.409	＜0.001
LDL (mmol/L) ∮	2.18(1.60~2.87)	1.68(1.03~2.47)	-6.541	＜0.001
DBI (umol/L) *∮	0.3(0.3~0.3)	0.3(0.3~0.3)	-6.283	＜0.001
IBIL (umol/L) *	9.30(5.69~14.81)	9.46(5.49~16.56)	-0.833	0.405
ALB (g/L) *∮	38.80(34~43)	34.96(27.99~39.50)	-7.761	＜0.001
GLO (g/L) *∮	32.65(29.47~36.55)	31.08(26.55~35.19)	-4.473	＜0.001
A/G*∮	1.16(1.00~1.33)	1.08(0.89~1.29)	-3.946	＜0.001
AST (U/L) *∮	30.06(23.36~44.30)	72.11(34.93~220.44)	-12.443	＜0.001
ALT (U/L) *∮	26.85(18.46~45)	44.49(21.51~125.27)	-6.694	＜0.001
OSM (mOSM/L) *∮	270.48(264.56~276.23)	279.07(269.78~286.82)	-8.792	＜0.001
**Blood gas**				
pH	7.43(7.40~7.46)	7.235(7.08~7.41)	-14.000	＜0.001
PCO_2 (mmHg) *∮	32(28~37)	34(25~48)	-2.446	0.014

PaO_2 (mmHg) *∮	102.5(81~132)	85.5(60~119)	-6.350	＜0.001
Na^+(mmol/L) *∮	134(131~136)	135(130~139)	-1.957	＜0.001
K^+(mmol/L) *∮	3.8(3.50~4.18)	4.6(3.80~5.28)	-10.157	＜0.001
Ca^{2+} (mmol/L) *∮	1.1(1.05~1.14)	1.05(0.99~1.12)	-6.492	＜0.001
HCO^- (mmol/L) *∮	21.42(19~24)	16.21(12.33~19.60)	-13.211	＜0.001
LAC (mmol/L) *∮	1.5(1.10~2.20)	6.4(3.00~11.08)	-18.246	＜0.001
L/A*∮	0.04(0.03~0.06)	0.18(0.09~0.35)	-18.698	＜0.001
**Coagulation indices**				
PT (s) *∮	12.4(11.5~14)	14.8(12.7~18.3)	-9.967	＜0.001
PTA (%)*∮	82.21(62.50~97.85)	61.65(41.07~79.31)	-9.004	＜0.001
INR*∮	1.10(1.02~1.25)	1.3(1.1~1.63)	-8.358	＜0.001
FIB(g/L) *∮	3.49(2.88~3.95)	3.23(2.17~3.93)	-4.264	＜0.001
APTT (s) *∮	31.3(28.7~33.9)	33.7(29.1~38.8)	-5.549	＜0.001
TT (s) *∮	19.8(18.5~21.58)	21.3(19.1~23.9)	-6.199	＜0.001
D-dimer (ng/ml) *∮	296.5(117.5~876.5)	2711(863~8420)	-16.157	＜0.001
**Inflammatory factors**				
CRP (mg/L) *∮	13.15(5.93~33.90)	42.25(9.75~82.50)	-7.340	＜0.001
IL-6 (pg/ml) *∮	11.33(5.02~39.25)	217.7(56.24~972.78)	-17.835	＜0.001
PCT (ng/ml) *∮	0.07(0.04~0.21)	0.51(0.10~9.41)	-10.993	＜0.001
**Cardiac markers**				
cTnI (ng/L) *∮	0.014(0.012~0.044)	0.136(0.020~1.500)	-10.909	＜0.001
CK-MB (U/L) *∮	1.09(0.65~2.09)	3.65(1.27~15.22)	-10.756	＜0.001
BNP (ng/L)* ∮	400.5(78.33~2062.50)	3010(459.25~7236.00)	-9.890	＜0.001

*Data are presented as median (interquartile range); Mann-Whitney U test was used for comparison. # Indicates that categorical variables were expressed as frequencies and percentages and analyzed by the chi-square test or Fisher's exact test. ∮Difference between groups is statistically significant ($P < 0.05$).

MAP is mean arterial pressure; WBC is white blood cell; NEU is neutrophil count; LYM is lymphocyte count; RBC is red blood cell count; HCT is erythrocyte pressure volume; HB is hemoglobin; PLT is platelet count; BUN is urea; Cre is creatinine; GLU is glucose; Lactic acid is venous lactic acid; CHO is total cholesterol; LDL is low-density lipoprotein; DBI is direct bilirubin; IBIL is indirect bilirubin; ALB is albumin; GLO is globulin; A/G is albumin/globulin ratio; AST is aspartate aminotransferase; ALT is alanine aminotransferase; OSM is osmolality; pH, PCO_2 is partial pressure of carbon dioxide; PaO_2 is partial pressure of oxygen in arterial blood; Na^+ is sodium; K^+ is potassium; Ca^{2+} is calcium; HCO^- is carbonic acid Na^+ for sodium ions; K^+ for potassium ions; Ca2+ for calcium ions; HCO^- for bicarbonate ions; LAC for arterial blood lactate, L/A for lactate/albumin ratio; PT for prothrombin time; PTA for prothrombin time activity; INR for International Normalized Ratio; FIB for fibrinogen; APTT for Activated Partial Thromboplastin Time; TT for Thromboplastin Time; D-dimer for D-dimer; CRP is C-reactive protein; IL-6 is interleukin-6; PCT is procalcitonin; cTnI is troponin I; CK-MB is creatine kinase isoenzyme; BNP is B-type natriuretic peptide.
